# Active legs: Impact of physical activity as an adjuvant treatment in the healing of venous ulcers in primary care: a RCT protocol study

**DOI:** 10.1186/s12912-023-01214-y

**Published:** 2023-03-10

**Authors:** Borja Herraiz-Ahijado, Carmen Folguera-Álvarez, José Verdú-Soriano, Pilar Mori-Vara, Milagros Rico-Blázquez

**Affiliations:** 1 Sanchinarro Healthcare Center. Primary Care Assistance Management, Madrid Health Service, Calle San Martín de Porres, 6., Madrid, 28035 Spain; 2 Research Network on Chronicity, Primary Care and Health Promotion (RICORS-RICAPPS), Instituto de Salud Carlos III, Madrid, Spain; 3 Gregorio Marañon Health Research Institute, Madrid Health Service, Madrid, Spain; 4 Nursing Department. Faculty of Nursing, Physiotherapy and Podiatry, Universidad Complutense de Madrid, Madrid, Spain; 5 PhD student. Doctoral Program in “Cuidados en Salud”, Universidad Complutense de Madrid, Madrid, Spain; 6 La Paz Healthcare Center. Primary Care Assistance Management, Madrid Health Service, Rivas-Vaciamadrid, Spain; 7 Research Group on Public Health - Lifestyles, nursing methodology and care in the community environment, Universidad Complutense de Madrid, Madrid, Spain; 8 Faculty of Health Sciences. Department of Community Nursing, Preventive Medicine, Public Health and History of Science, University of Alicante, Alicante, Spain; 9 Health Innovation Research Group. Nursing Department. Faculty of Nursing, Physiotherapy and Podiatry, Universidad Complutense de Madrid, Madrid, Spain; 10 Research Unit. Primary Care Assistance Management, Madrid Health Service, Madrid, Spain

**Keywords:** Venous ulcer, Wound healing, Exercise, Nursing, Primary health care, Quality of life, Aging

## Abstract

**Background:**

Venous ulcers usually present a torpid evolution with a negative impact on patients’ quality of life. In primary care, they account for 2.5% of nursing consultations and their treatment represents high costs for national health systems. These patients usually have a low level of physical activity, with muscle pump dysfunction of the lower limbs, which may improve with increased physical activity. The purpose of this study is to analyse the effectiveness of a structured intervention involving physical activity and exercise (Active Legs) as an adjuvant treatment in improving healing of chronic venous ulcers at 3 months follow-up.

**Methods:**

A randomized, multicentre clinical trial. A total of 224 individuals receiving primary nursing care with a diagnosis of venous ulcer, with a diameter of 1 cm or greater and an ankle-brachial index between 0.8 and 1.3, able to comply with the study requirements and consenting to participate, will be sequentially included (112 per group).

Both groups will receive the standard treatment in primary care, with cleansing, debridement and healing in a moist environment together with multilayer compression therapy. The intervention group will also receive a structured educational intervention involving lower limb physical exercise and daily ambulation guidelines.

The primary response variables will be complete healing –understood as complete and sustained epithelialisation for at least 2 weeks– and time to healing. The secondary variables will be degree of healing, ulcer area, quality of life, pain and variables related to the healing process, prognosis, and recurrences. Sociodemographic variables, adherence to treatment and satisfaction variables will also be recorded. Data will be collected at baseline, at 3 months and at 6 months follow-up. Survival analysis (Kaplan-Meier and Cox) will be performed to measure primary effectiveness. Intention-to-treat analysis.

**Discussion:**

If the intervention is effective, a cost-effectiveness analysis could be conducted and implemented as an additional intervention in the usual venous ulcer treatment in primary care.

**Trial registration:**

NCT04039789. [https://ClinicalTrials.gov]. 07/11/2019.

**Supplementary Information:**

The online version contains supplementary material available at 10.1186/s12912-023-01214-y.

## Background

Ulcers of venous aetiology are lesions caused by a process of chronic venous insufficiency [1, 2], with a tendency to a torpid evolution, a low tendency to spontaneous healing [1, 3] and a high recurrence rate [4, 5]. For diagnosis, in addition to the presence of signs of chronic venous insufficiency, it is necessary to assess the ankle brachial index (ABI) [2].

In Spain, lower limb ulcers have a prevalence of 0.1 to 0.3% in the adult population [2] and an incidence of 3 to 5 new cases per thousand people annually (2.6), both values becoming twice as high in the over-65 population segment [2]. Venous ulcers (VU) account for 75–80% of all lower limb ulcers [2, 6] with an average duration of 10.7 months (+ − 27 months) and over 50% being recurrent ulcers [1, 2]. They represent 2.5% of primary care consultations and between 1.5 and 3% of total healthcare expenditure in neighbouring countries [1].

In clinical terms, ulcers of venous origin usually show signs such as high levels of exudate, malodour, moderate pain, and infection, causing sleep disturbances, mobility problems, decreased vitality and even dependence in performing basic activities of daily living [7–9]. A negative emotional impact and a low perceived quality of life have also been observed [10, 11].

There are two main components to ulcer treatment. On the one hand, the topical healing of the wound, favouring a moist environment through the use of dressings [1], and on the other hand, the control of chronic venous insufficiency through the use of compression therapy [1, 12].

Recent studies show that these patients suffer from muscle pump dysfunction of the lower limbs [13, 14], resulting in poorer healing rates [5] and decreased ankle mobility [15, 16] associated with a process of fibrosis involving the Achilles tendon [16].

The lack of competence of the muscle pump, together with the decreased ankle mobility and the age at which VUs are most prevalent (over 65 years) could be among the reasons why approximately 50% of patients suffering from VU have an insufficient level of physical activity (less than 10 min of ambulation per day), as suggested by various authors [5, 7, 13, 17].

In recent years, the published studies have demonstrated the positive effects of the daily practice of an exercise and daily ambulation program at home on the functioning of the calf muscle pump [5, 17, 18], the improvement in ankle mobility [16, 19], the significant increase in tissue perfusion parameters [4, 11, 14] and the improvement of more active lifestyle behaviours among patients [7]. Two systematic reviews published in 2018 with a total of 405 patients recommend the use of aerobic and resistance exercise in the treatment of venous ulcers [20, 21].

With regard to the type of exercises to be performed, there is evidence that a standing position on tiptoe, as well as the flexion-extension of the feet in a seated position, effectively stimulate the calf muscle pump and improve venous return [14, 17, 22]; lower limb resistance exercises also showed benefits in muscle pump functionality and ankle mobility [16, 23] while daily ambulation is also effective as it allows for increased blood pumping from the leg to the heart [11, 14, 17], along with an improvement in the leg muscle pump due to the movement of the ankle joint [5, 14, 16].

Most of the revised studies have been conducted in the United Kingdom, Poland, Brazil, Australia, the Netherlands, and the United States, with small sample sizes. We have not found scientific evidence in Spain on the study of the influence of exercise on the healing of venous ulcers, which justifies the need to conduct this trial, the aim of which is to compare the effectiveness of a structured educational intervention in physical exercise versus the standard practice in the healing of venous ulcers.

## Methods/design

### Main objective

To evaluate the effectiveness of the “Active Legs” intervention as an adjuvant treatment to standard primary care management of venous ulcers for improving the rate of complete healing at 3 months follow-up compared with the usual practice.

### Secondary objectives

To determine whether the Active Legs intervention produces better results than the standard practice on the rate of venous ulcers with complete healing at 6 months follow-up, the degree of healing achieved, the number of recurrences, the quality of life and the pain, as well as to describe the degree of adherence to and satisfaction with the intervention.

#### Design and setting

A pragmatic, randomized, multicentre, open-label, clinical trial of two parallel groups with 6-month follow-ups. The SPIRIT checklist are available as supporting information (see Additional file 1).

#### Setting and study population

The study will be carried out in 16 primary care health centres in the Region of Madrid.

It will include participants aged 18 or older, with a diagnosis of venous ulcer recorded in the electronic medical record (ABI greater than 0.8 and less than 1.3; diameter of the lesion greater than or equal to 1 cm) and under treatment in primary care nursing consultations. The individuals must be able to walk with or without the aid of devices, understand and answer the questionnaires autonomously, be accessible throughout the duration of the study and have expressed their agreement to participate and signed the consent form.

Those who are unable to commute to the health centre, or who reside outside the area where the research is carried out for more than 6 months per year, will be excluded. People with mixed ulcers, deep vein thrombosis (DVT) in acute phase, decompensated heart failure, dermatitis in acute phase, rheumatoid arthritis, undergoing treatment with antineoplastic drugs or with some absolute contraindication for physical exercise will also be excluded.

Withdrawal criteria are set for patients who, during the course of the trial, present a change in their clinical condition that prevents them from further participation, such as inflammation of the locomotor system (with heat, flushing, pain and functional impotence) or trauma due to a fall during the course of program with or without fracture and/or haematoma at both joint and soft tissue level (muscle and tendons) [24], must drop out of the study.

### Randomization and masking

Simple random allocation to one of the two study groups via the Electronic Data Collection Notebook.

It is not possible to mask the intervention in this type of trial, although the analysis will be performed by professionals who are unaware of the allocation.

### Sample size

Previous studies have obtained 50% complete healing rates at 12 weeks follow-up. Sample size has been calculated considering a relevant post-intervention average difference of 20% of complete healing between the intervention and control groups. Assuming an alpha error of 0.05 and a beta error of 0.02, 93 patients are necessary for each group. Expecting a 20% loss to follow-up after a 3-month period, the required sample size is 224 patients (112 per group).

### Recruitment

Study enrolment will be offered during the usual nursing practice, when eligible patients attend for follow-up wound care. They will be sequentially recruited upon signing the consent form.

#### Interventions

The **control group** will receive the usual treatment following the recommendations for the treatment of skin ulcers of the Madrid Health Service [1], which consists of assessment, cleansing, antisepsis, debridement, topical treatment (healing in a moist environment through the use of dressings) and multilayer compression therapy.

**Fig. 1:**
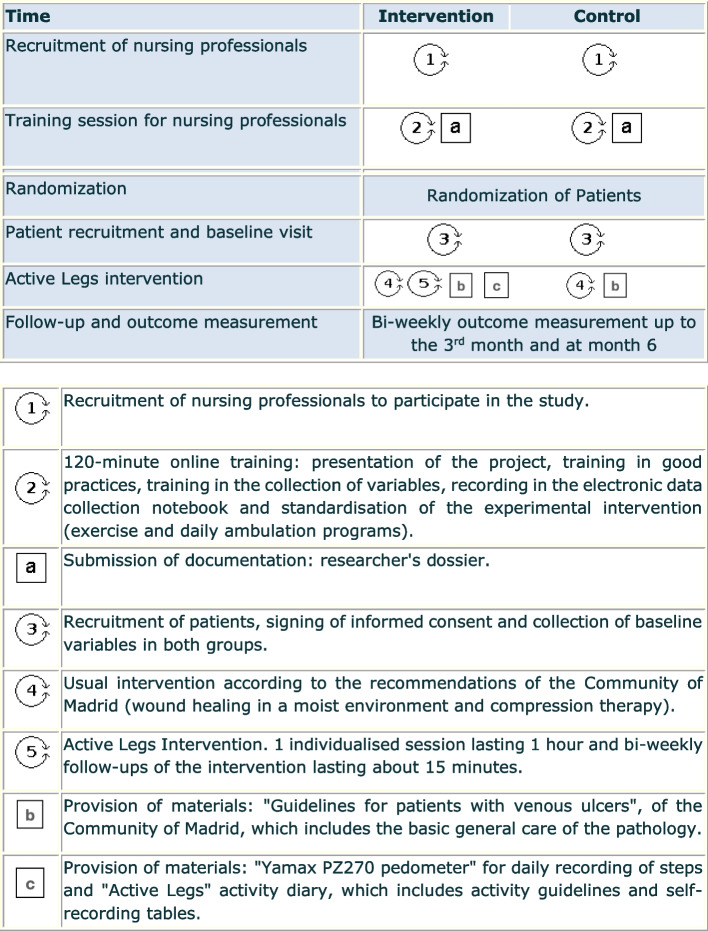
Components of Active Legs complex intervention vs standard clinical practice

The **intervention group** will receive the usual treatment together with the “Active Legs” intervention, which consists of an evidence-based, structured, nurse-led educational intervention in health centre consultations. It incorporates a home-based lower limb exercise program and daily ambulation guidelines. Figure 1 shows schematically the different components of both the control and the Active Legs interventions. The home lower limb exercise program consists of four exercises of progressive difficulty, which the patient will perform twice a day, at least 5 days a week. In addition, patients will be required to follow a program of progressive daily ambulation until the target of 150 min/week (30 minutes for 5 days a week) is reached [25]. The detailed intervention is described using TIDieR (see Additional file 2).

Patients will be provided with an “Active Legs” diary containing all the information on the exercise program and a pedometer to record daily steps.

Those who have not reached complete healing at the end of the study will continue with the same treatment until complete healing is achieved and may resume the daily ambulation and exercise program.

In order to avoid possible differences in the application of the Active Legs program, training has been planned beforehand for the nursing professionals participating in the study, including both training in injury assessment and educational intervention.

### Outcomes

**The primary outcome** will be complete healing at 3 months follow-up: (yes/no), where complete healing is defined as complete epithelialisation maintained for at least 2 weeks and the time (in days) between the start of the study and complete wound healing.

**Secondary variables** are defined as complete healing at 6 months follow-up (yes/no), the degree of healing (Resvech 2. 0) which consists of 6 dimensions (depth, size, edges, wound bed, exudate and signs and symptoms of infection) with ascending scoring scales based on the severity of the dimension studied and a total score ranging from 0 to 35 [26]; the ulcer area (cm2), measured by digital photography and subsequent image processing using the open source Java image processing program “The ImageJ ecosystem” [27]; the VAS scale for perceived pain [28]; the level of adherence to the “Active Legs” intervention measured by the combined variable number of steps and time by means of the pedometer record (Yamax PZ270) and patients’ self-reported information on the home exercise program by means of the activity diary and the health-related quality of life measured with the CCVUQ-e, a questionnaire which in addition to an overall synthetic quality of life score, assesses the following dimensions: pain, depression, social relationships, impairment in performing activities of daily living and body image, with a score of 22 to 112 [29].

**Variables related to the healing process** are defined as Body Mass Index (kg/cm2); baseline pathology, diabetes mellitus (yes/no); last glycosylated haemoglobin (HbA1) value in the EHR; ABI score; tobacco and alcohol consumption; topical treatments used in existing ulcers; systemic treatments; adherence to multilayer compression therapy (yes/no); and physical activity level measured by the Minnesota Leisure Time Physical Activity Questionnaire, short version [30] and type of daily ambulation.

**Prognostic variables** are defined as location of the ulcer at the time of the study, number of ulcers at the time of the study, time (in days) of evolution of the venous ulcers before inclusion in the study and whether they are recurrent ulcers (yes/no).

**Variables related to recurrence (measured at 6 months follow-up)** are defined as occurrence of recurrence (yes/no), use of compression stockings (yes/no), light/normal/strong compression and hydration of the legs (yes/no).

**Additionally, data such as age, sex,** living alone (yes/no), employment status and level of education are collected.

### Participant timeline

Table 1 shows the participant timeline.

**Table 1:**
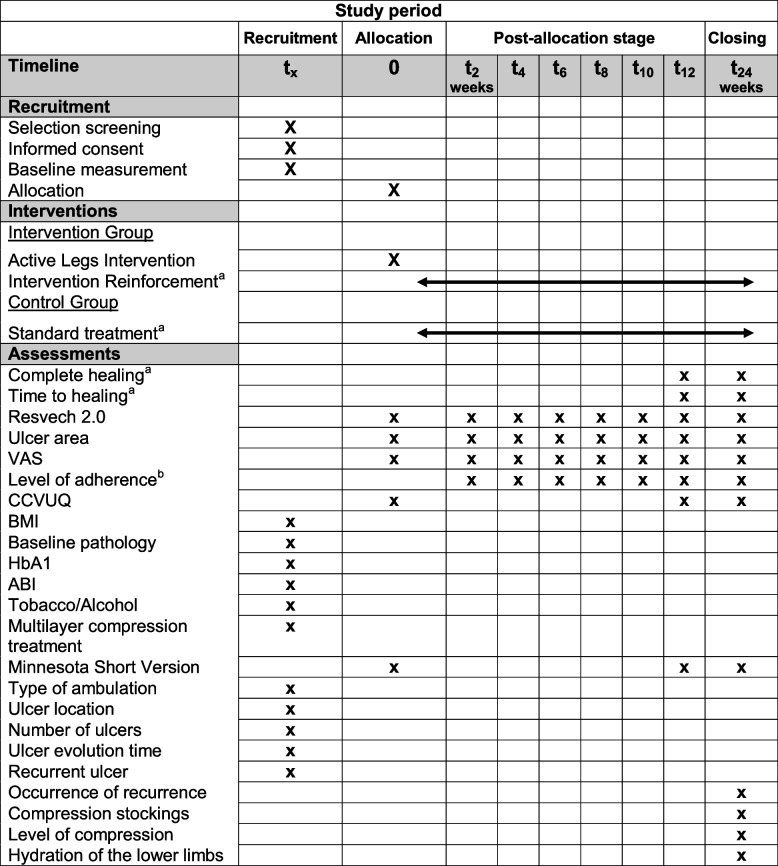
Participant timeline

^a^Until complete healing of the wound

^b^Intervention group only

### Data collection methods

Patient information will be obtained through clinical interviews, electronic medical records and wound examination in the nursing consultation of the professionals participating in the study. The same information will be collected from participants in the control and intervention groups, except for the level of satisfaction and adherence. Data will be collected every 14 days for the first 3 months or sooner if the ulcers have healed, and at 6 months follow-up.

Patients who refuse to participate in the study, losses and dropouts and their cause, as well as patients who are required to drop out because of withdrawal criteria, will be recorded.

### Data management

The information will be recorded in an electronic data collection notebook designed for the study, which contains all the questionnaires and scales, collected in the variables section, for implementation. Photographs of ulcers will be temporarily stored in the electronic notebook until the ulcer area is measured.

### Losses in participation

Losses and dropouts during the study and their cause, as well as patients who are required to drop out because of withdrawal criteria, will be recorded. In order to minimise potential losses, as a method of encouraging participation, patients and/or carers will be contacted by telephone to arrange visits.

In the event of dropouts during follow-up, at least 2 telephone calls will be made to record their cause.

### Analysis

A descriptive analysis (means, medians, frequencies of distribution) will be performed. The results for the primary outcomes will be analysed on an intention-to-treat (ITT) basis. To analyse the principal effectiveness the incidence rates of completely healed ulcers will be compared with their point estimate and 95% confidence interval. Time to complete healing will be compared using Kaplan-Meier curves (Log-rank test). A Cox regression model will be used to adjust for prognostic factors.

To analyse secondary outcomes, an explanatory model will be fitted with linear or logistic analysis, as appropriate.

Significance will be set at *p* < 0.05. All analyses will be performed using STATA software.

## Discussion

The aim of the study is to measure the effectiveness of a structured educational intervention on lower limb physical exercise and daily ambulation (Active Legs) as an adjuvant treatment to topical treatment and compression therapy addressed to individuals with venous ulcers undergoing treatment in primary care.

This research addresses a health problem that affects people of all ages, although its prevalence increases with age, being much more frequent in people over 65 years of age [2].

Venous ulcers can aggravate the prognosis of other pathologies, cause pain, and have a negative impact on patients’ wellbeing, self-perception, and perceived quality of life [10, 11]. They also limit the ability to manage daily self-care and impair mobility, thus being a risk factor for dependency and the onset of early frailty [10]. *Research on frailty* is a priority due to the increasing elderly population in many countries, including Spain. Institutions such as the World Health Organisation in its World Report on Ageing and Health [31] stress that population ageing requires a comprehensive public health response, with the development of policies that emphasise the need for healthy and active ageing.

Incorporating a routine of basic lower limb exercises at home and daily ambulation into the self-care of older people with venous ulcers could help them not only to improve wound healing, but also to have a more active and autonomous lifestyle, improving their self-perception, self-concept, and perceived quality of life [7].

This clinical trial follows a pragmatic approach, very close to routine clinical practice, which uses the capabilities of the National Health System. It will be carried out at the primary care level, with the basic resources and means usually available in health centres and does not require additional training of nursing professionals. The study proposes the use of a pedometer as an objective measure of adherence, but this resource would not be necessary for the implementation of the active legs’ intervention in clinical practice.

Given that the research team is directly linked to the healthcare activity within the health system, and that the strategic lines of the organisation have seen the need to protocolise the care of venous ulcers in a Protocol for the treatment of skin ulcers in the Community of Madrid, if the intervention were effective it would be easily transferable and its cost-effectiveness and impact on the consumption of healthcare resources could be analysed in the medium term.

The result will in turn lead to a reduction in the cost of treatment, optimising the health system’s human and material resources. At the same time, it will contribute to homogenise the intervention to be developed for these patients, favouring equity.

Patients will be recruited by their own reference nurses. This may increase variability, which we will try to minimize by training all participating nurses beforehand and by protocolizing the research, implementing an eDCN specifically designed for the study and providing supporting graphic documentation.

This study, therefore, proposes, through nursing intervention at the primary care level, to address the management of venous ulcers from a different perspective, focusing on the baseline aetiology of the condition as an adjuvant treatment and promoting an active lifestyle to benefit patients’ overall health (biopsychosocial model).

## Supplementary Information

**Additional file 1.**

**Additional file 2.**

## Data Availability

Not applicable.

## Abbreviations

AVH: Ambulatory Venous Hypertension
VU: Venous ulcer
CCVUQ: Charing cross venous ulcer questionnaire
VAS: Visual analogue scale
ABI: Ankle brachial index (ABI)
DVT: Deep vein thrombosis
PCAM: Primary Care Assistance Management
eDCN: Electronic Data Collection Notebook
EHR: Electronic Health Record
CREC: Clinical Research Ethics Committee
