# Supplementary material for: Active legs: Impact of physical activity as an adjuvant treatment in the healing of venous ulcers in primary care: a RCT protocol study
Source: BMC Nurs. 2023 Mar 10;22:65. doi: 10.1186/s12912-023-01214-y (PMC9999587; doi:10.1186/s12912-023-01214-y)
Supplement: Supplementary file 2 — Additional file 2. [file 12912_2023_1214_MOESM2_ESM.pdf]

The TIDieR (Template for Intervention Description and Replication) Checklist*:

Information to include when describing an intervention and the location of the information

Item number	Item	Where located ** Primary paper (page or appendix number)	Other † (details)
	**BRIEF NAME**		
**1.**	"Active Legs": structured educational intervention of lower limb exercise	Page 1	
	**WHY**		
**2.**	Since there is evidence that daily physical activity improves venous return in the lower limbs and that the aetiology of vascular ulcers is venous insufficiency, we can assume that a more active lifestyle could influence the evolution of this type of wounds. The main objective of the "Active Legs" study is to evaluate the effectiveness of the intervention as an adjuvant treatment to the standard management of individuals suffering from venous ulcers in primary care in order to improve the rate of complete healing at 3 months follow-up compared to the usual practice in a clinical trial.	Pages 2,3,4	
	**WHAT**		
**3. y 4.**	**Materials and Procedures**: **Intervention group:** This is an evidence-based educational intervention, structured and led by nurses in primary care consultations. Patients are instructed in the use of a pedometer as a useful tool to increase daily physical activity and as a daily record of steps. In addition, they are given a notebook containing the exercises to be practised and general information on the care of chronic venous insufficiency, providing a detailed explanation of the exercise routine to be carried out progressively at home and also as a support for the daily record of patients' own performance. The home program consists of 3 lower limb exercises of progressive difficulty and graded intensity, following by standing up and sitting down from a chair with the same	Pages 6,7	Figure 1

	frequency and intensity as the previous exercises. Daily walking should be started at a moderate pace of 10 minutes a day, and this time should be progressively doubled until the target of 150 min/week (30 minutes for 5 days a week) is reached.<br><br>**Control group:** Standard practice includes treatment for venous ulcers according to the recommendations of the Community of Madrid, consisting of assessment, cleansing, antisepsis, debridement, topical treatment (healing in a moist environment through the use of dressings) and multilayer compression therapy.		
	**WHO PROVIDED**		
**5.**	Primary care nurses with experience in venous ulcer management and 6 hours of online basic training on the project in order to minimise differences in program implementation as much as possible.	Page 7	
	**HOW**		
**6.**	The intervention is applied in a face-to-face and individualised manner in nursing consultations every two weeks, following the protocol and in conjunction with wound care treatment. For patients with poor adherence and recurrence assessment, the intervention can also be carried out by telephone	Pages 6,7	
	**WHERE**		
**7.**	The program is delivered at patients' homes, with no equipment required.	Pages 6,7	
	**WHEN and HOW MUCH**		
**8.**	The lower limb exercise program is performed by the patient twice a day, five days a week, with an estimated duration of 10 minutes per session and daily ambulation for 30 minutes per day, five days a week.	Page 7	

	**TAILORING**		
**9.**	As patients with this pathology have low levels of physical activity, the lower limb exercise program has four levels of graded intensity in which, if level 1 is successfully completed for three days, progress to the next level will be made. In addition, daily ambulation will be progressive, starting with 10 minutes a day and progressively increasing until the target (30 minutes) is reached.	Page 7	
	**MODIFICATIONS**		
**10.**	N/A		
	**HOW WELL**		
**11.**	Planned: The variable adherence to the intervention was measured by means of patients' self-recording in their notebooks of the exercises and steps completed. These data were categorised as: Excellent (75% or more of the prescribed sessions), Good (between 50-74%), Moderate (between 25-49%) and Poor (less than 25%). For patients with 50% or less, a booster session is scheduled (up to a maximum of 2 booster sessions).	Page 8	
**12.**	Actual: N/A		
